# Intrauterine contraceptive discontinuation reasons among female trial participants living with HIV in Cape Town, South Africa: A qualitative analysis

**DOI:** 10.3389/fgwh.2023.1010794

**Published:** 2023-03-24

**Authors:** Subarna Pradhan, Elizabeth E. Tolley, Nontokozo Langwenya, Heidi E. Jones, Donald R. Hoover, Gregory Petro, Landon Myer, Catherine S. Todd

**Affiliations:** ^1^ Global Health, Population, and Nutrition Department, FHI 360, Durham, NC United States; ^2^ Division of Epidemiology and Biostatistics, School of Public Health and Family Medicine, University of Cape Town, Cape Town, South Africa; ^3^ Department of Epidemiology and Biostatistics, City University of New York School of Public Health, New York, NY, United States; ^4^ Department of Statistics and Institute for Health Care Policy and Aging Research, Rutgers University, Piscataway, NJ, United States; ^5^ Department of Obstetrics and Gynaecology, University of Cape Town and New Somerset Hospital, Cape Town, South Africa

**Keywords:** HIV, contraception, long-acting reversible contraception, intrauterine device, discontinuation, bleeding side-effects

## Abstract

**Introduction:**

While young women in South Africa may navigate both HIV infection and pregnancy risks, intrauterine contraceptive (IUC) use is low. Though IUCs have low failure rates, concerns exist about whether the IUC is an appropriate method choice for women living with HIV (WLHIV). In this qualitative study, we explore WLHIVs' experiences of using IUC and reasons for discontinuation.

**Methods:**

This qualitative study included in-depth interviews (IDIs) with 17 WLHIV who electively discontinued their allocated IUC while participating in a randomized controlled trial comparing the safety of the levonorgestrel intrauterine system (LNG-IUS) and the copper intrauterine device (C-IUD) in Cape Town, South Africa. The transcripts were coded and emergent themes were grouped to examine women's experiences with IUC use and reasons for discontinuation, highlighting experiential differences between the two methods.

**Results:**

Women's experience with the allocated IUC related most commonly to bleeding and/or abdominal pain just after insertion. Most C-IUD discontinuers, but only one LNG-IUS discontinuer, complained of pain and/or increased bleeding as the main reason for removal. Three women (two LNG-IUS, one C-IUD) requested removal because they desired pregnancy, while two others discontinued due to fears the IUC was exacerbating non-gynecologic conditions (hypertension, diabetes). Generally, women acknowledged advantages of IUC use and many expressed their desire to continue use if not for the side effects.

**Conclusions:**

In the South African context, IUC use for WLHIV should be promoted with specific counseling considerations. Both providers and potential users should receive specific information about potential menstrual-related side effects and countering common misperceptions to enable informed contraceptive decision-making.

## Introduction

Intrauterine contraceptives (IUCs), including the copper intrauterine device (C-IUD) and levonorgestrel intrauterine system (LNG-IUS), are highly effective, long-acting reversible contraceptive (LARC) methods that many women rely upon globally to achieve desired family size (1, 2). However, as with all contraceptives, there are advantages and disadvantages regarding convenience, efficacy, and side-effects and the relative weighting of these aspects for method choice and continuation is highly individual (3).

IUCs are frequently associated with bleeding symptoms, ranging from menorrhagia to amenorrhea (4–6). In a secondary analysis of 1,947 Chilean C-IUD users, pain (38%) and bleeding (68%) declined gradually, peaking at nine weeks, but remained substantial, with 33% and 48% of women reporting these symptoms, respectively, after 39 weeks (4). Many women requesting C-IUD removal did not differ by perceived severity of pain or bleeding from those continuing the C-IUD (4). The LNG-IUS is associated with reduced menstrual pain and bleeding, often resulting in amenorrhea or irregularity (5). Among the USA CHOICE cohort, 7% of 1,925 women selecting an IUC discontinued within six months, with no statistically significant difference by IUC type. Cramping was the most common discontinuation reason for LNG-IUS (28%) and C-IUD (35%), followed by bleeding symptoms (9% and 19%, respectively) (6).

IUCs are relatively underutilized by women living with HIV (WLHIV) for myriad reasons, despite documented safety profiles regarding HIV disease progression and pelvic infection (7–9). Based on perceived bleeding pattern alterations by IUCs, WLHIV may have additional concerns related to bleeding changes associated with HIV status (10, 11); surrounding antiretroviral therapy (ART) interactions; or perceived increased risk of disease transmission, which may limit IUC use or increase likelihood of discontinuation. Among WLHIV cohorts using IUCs, discontinuation rates vary widely and reasons provided for discontinuation are similar to those reported by HIV-negative women (7–9, 12, 13). However, there has been little exploration of why WLHIV discontinue IUCs and whether and to what degree HIV disease influences discontinuation decisions.

The Comparison of Two IUDs among Cape Town HIV-positive Women: A Randomized Controlled Trial Assessing Safety of Registered Products in South Africa (2IUDnCT) randomized controlled trial was conducted in Cape Town, South Africa between October 2013 and July 2018 to compare IUC safety and acceptability among WLHIV. During this period, HIV treatment expanded from ART initiation based on lymphocyte count to initiation at diagnosis (14). This qualitative sub-study was conducted among women requesting IUC removal during study follow-up to explore women's experiences with IUC use and contextualize discontinuation reasons. Where possible, differences in user experiences are highlighted between the LNG-IUS and C-IUD to inform provider and patient education and counselling tools for IUC use among WLHIV.

## Materials and methods

### Parent study and qualitative sub-study overview and eligibility criteria

The 2IUDnCT trial (ClinTrials.gov: NCT01721798) enrolled 199 WLHIV in Gugulethu, Cape Town, South Africa between October 2013 and December 2016. Study methods are described in detail elsewhere (14). Eligibility requirements among WLHIV aged 18 to 40 years included being at least six months postpartum, not desiring pregnancy in the next 30 months, willingness to be randomized to IUC type, and being either virally suppressed on ART or not yet eligible for ART by provincial guidelines. Consenting women were randomized 1:1 to receive the LNG-IUS or C-IUD with both participant and study staff (with exception of study nurse and manager) masked to IUC type and followed up to 24 months to assess IUC safety and acceptability. Women could have their IUC removed on request and were retained in the trial following discontinuation. For this qualitative component of the study, only women who requested elective IUC removal during the study (*n* = 34) were eligible to participate in semi-structured in-depth interviews (SSI) to explore IUC experiences and removal reasons. The study was approved by FHI 360's Protection of Human Subjects Committee and the Human Research Ethics Committee at the University of Cape Town Faculty of Health Sciences.

### Measures

The SSI guide was developed, translated into isiXhosa, and pre-tested among volunteers using an IUC who were not study participants. The interview guide included questions on general IUC perceptions and whether women attributed changes in menstruation or HIV disease to the IUC. Women were then asked about health maintenance following HIV diagnosis, condom use decision-making, and male partner feelings about IUC. The interviewers further focused on discontinuation by asking participants to rank reasons from most to least important and also explored themes of impact of side-effects on lifestyle and balance between tolerability of effects relative to perceived contraceptive efficacy. Finally, the interviews explored ideal contraceptive method features and reasons for selecting a specific method after IUC removal.

### Data collection

Women requesting IUC removal were informed of the qualitative sub-study prior to removal and invited to participate in a 30 to 60-minute interview, scheduled at their convenience. Following examination, IUC removal, and counselling and potential provision of a new method, interested women went to a private room and reconfirmed informed consent for the SSI. Female isiXhosa-speaking staff trained in qualitative techniques conducted the SSIs. Following the interview, participants were offered counselling and scheduled for their next study appointment. Some women were inadvertently notified of IUC type prior to SSI but unmasking was not recorded in study instruments.

### Qualitative data management and analysis

Interviews were audio-recorded, transcribed and translated into English, and uploaded into NVivo 12 (QSR International Pty Ltd. Version 12, 2018). A four-step thematic analysis process (i.e., reading, coding, data display, and data reduction) was followed (15). Two independent researchers (S.P. and E. T., not masked to IUC type) first reviewed several transcripts to identify emergent themes that should be considered for more in-depth analysis. Second, the researchers shared and discussed potential codes to develop a common codebook. The coding scheme was applied to two new transcripts to ensure they had the same understanding of the codebook. The researchers identified any discrepancies in which codes were applied and determined whether any codes or their definitions required refinement. Remaining transcripts were divided between researchers and independently coded. As a third step, both researchers read through assigned coding reports, developing memos describing code/theme dimensions (e.g., for “bleeding” sub-code, the memo grouped and described the range of bleeding, spotting, and concurrent non-bleeding experiences reported across interviews), and identifying exemplar quotes. Finally, the researchers developed matrices to note presence or absence of coding content to identify emergent patterns between IUC types (15).

## Results

Overall, 34 (30 C-IUD, four LNG-IUS) women requested elective IUC removals. Of these 34, 17 women (13 C-IUD and four LNG-IUS users) agreed to participate in this qualitative sub-study. Of the other 17 women approached who declined interview, time constraints were the most common reason for declining.

### Participant characteristics

All participants had children, with median parity of 2.0 births (Table 1). Participants were experienced contraceptive users, reporting prior injectable progestin or condom use at enrolment. The median time participants used the IUC from insertion to removal was 7.75 months, ranging from 1.5–21.25 months.

**Table 1:** In-depth interview participant characteristics & main reason for intrauterine contraceptive (IUC) discontinuation among women living with HIV participating in a clinical trial in Cape Town, South Africa.

Characteristics	All	C-IUD	LNG-IUS
	(*n* = 17)	(*n* = 13)	(*n* = 4)
Age in years (median)	31.0	31.0	31.5
Number of children (median)	2.0	2.0	2.5
Employed, part-time or full-time, % (*n*)	11.7 (2)	7.6 (1)	25.0 (1)
Aware of partner’s HIV status, % (*n*)	52.9 (9)	46.1 (6)	75.0 (3)
Had regular partner at time of IUC discontinuation, % (*n*)	88.2 (15)	92.3 (12)	75.0 (3)
Contraceptive method reported at enrollment, % (*n*)	100 (17)	100 (13)	100 (4)
Norethindrone enanthate	17.6 (3)	23.0 (3)	0.0 (0)
Depot medroxyprogesterone acetate	29.4 (5)	23.0 (3)	50.0 (2)
Male condom (alone or with another method)	52.9 (9)	53.8 (7)	50.0 (2)
Time with IUC before discontinuation (median; months)	7.75	7.5	11.6

C-IUD, copper intrauterine device; HIV, human immunodeficiency virus; IUC, intrauterine contraceptive; LNG-IUS, levonorgestrel intrauterine system; *n*, number.

### Reasons for discontinuation

The reasons for discontinuation and their relative importance as relayed in IDIs are displayed in Table 2. The most common and important IUC removal reason was pain. About two-thirds of the sub-study women said they experienced some pain with IUC use and more than half said this was the most important reason why they stopped using their IUC. While only one LNG user gave this reason, eight C-IUD participants did. Most women who discontinued because of pain described sharp, debilitating pain that made work, sleeping or sex difficult. A few women associated pain with increased menstrual bleeding. Some women remarked that menstrual cramping had doubled in duration, along with bleeding:

*I could not continue using it (C-IUD) because of lower abdominal pains and prolonged menstrual cycle…cramp the whole day and night. In fact, I can't sleep because of the pain. I have been having such pain since I inserted the loop…since day one.* (7069, C-IUD user)

**Table 2:** Matrix for dominant and all discontinuation reasons among women living with HIV participating in a clinical trial in Cape Town, South Africa (*n* = 17).

Reasons For Removal												
	Increased Menstrual Bleeding				Irregular or Reduced Bleeding		Other Side Effects				Unrelated to Product	
*ID*	*IUC arm*	*Heavy Bleeding*	*Frequent (>2/mon)*	*Prolonged bleeding*	*Spotting*	*No/less bleeding*	*Pain*	*Weight change*	*Cough*	*CD4 change*	*To get pregnant*	*Health reasons*
7024	C-IUD	1		1								
7031	C-IUD	1						1		2		
7054	LNG-IUS			2	2						1	
7069	C-IUD			2			1					
8023^a^	C-IUD			2			1	2	2			
8037^a^	LNG-IUS					2	1	2				2
8051^a^	C-IUD		2									1
8053^a^	C-IUD					2	1					
8059^a^	LNG-IUS					2					1	
8072^a^	C-IUD						1				2	
8075^a^	C-IUD	2	2				1					
8097^a^	C-IUD			1								
8100^a^	C-IUD			2			1					
8102^a^	LNG-IUS						1	2				
8119^a^	C-IUD	1		1			2					
8123^a^	C-IUD	2		2			2				1	
8126^a^	C-IUD						1					
Any Experience		5	2	8	1	3	11	4	1	1	4	2
Main Reason					0	0	9	1	0	0	3	1

1 = Mentioned as main reason; 2 = Mentioned as reason but not main reason; Empty cell = Not mentioned.

^a^ Antiretroviral therapy use at enrollment. C-IUD, copper intrauterine device; HIV, human immunodeficiency virus; ID, study identification number; IUC = intrauterine contraceptive; LNG-IUS, levonorgestrel intrauterine system.

For some women, the pain was not accompanied by menstrual bleeding, or did not appear to affect their usual cycles. More often though, women who discontinued because of pain described sharp, debilitating pain that made work, sleeping or sex difficult. Several women described the pain as continuous, while others reported more acute episodes. Some attempted treating the pain themselves, with oral analgesics or enemas, before seeing a provider or seeking removal.

*On the first day, I felt a pain on my right-hand side of my body. I thought I was going to menstruate. I went to sleep, the next morning it was gone. The pain came back again on the second day. I thought of the same again on that day. I went to sleep, and it was gone again the next morning*. (8102, LNG-IUS user)

*Once I have these* (abdominal) *pains, I would think I'm about to have my period. But then the period would not come. The most painful part would be the backside of my abdomen… I would try enemas, hoping the pains would go away, but they won't get any better. Instead I would be worse, so much so that I won't be able to do anything. Another thing I noticed is about one of my legs. My left leg started to give me difficulties, it got very painful…The main reasons to remove this IUD is because of the pains I feel in my body”.* (8075, C-IUD User)

Bleeding changes were also commonly experienced, but less frequently cited as the main discontinuation reason. All but three women described some bleeding pattern change. Many women reported increased bleeding quantity or duration, but only four reported bleeding as the main discontinuation reason (Table 2).

*I used to have a menstrual period of three days but now that I was using it, I would go up to six or seven days longer. The blood will be so heavy that pads do not withstand it and I would end up using diapers….Before I had the IUC, I had a normal period of three days…I would use one pad a day.* (8119, C-IUD user)

One woman mentioned that she did not like the non-stop spotting she experienced, though this was not cited as a discontinuation reason. By contrast, three women, of whom two were in the LNG-IUS arm, mentioned experiencing no or reduced bleeding. None of these women attributed their discontinuation to such changes. Indeed, one woman, who discontinued to get pregnant, reported being happy to have less bleeding:

‘*Before I inserted a loop, when I menstruated, I would take about five days. When I inserted a loop, I took about three days. So that makes me happy’. (8059, LNG-IUS user)*

Desiring pregnancy was mentioned infrequently as the main discontinuation reason. Several other women also suggested that their partners wanted another child, but that they were not in agreement, yet were removing the IUC to please their partner.

Other side effects were rarely mentioned as discontinuation reasons but were raised during the discussion of reasons for discontinuation as negative aspects of IUC use. Four noticed weight changes after IUC insertion, with one (LNG-IUS) reporting weight gain. Women reporting weight loss were C-IUD users and two attributed this to increased bleeding. However, none specifically stated that discontinuation was wholly for these reasons but that these side effects added further impetus behind the decision to discontinue.

### Perceived IUC impact on HIV disease

When specifically asked whether they perceived any change in their HIV disease course following IUC use, participants rarely attributed IUC use to negative effects on their HIV status, with most noting no difference. One C-IUD user suggested the IUD might have caused her weight loss and reduced CD4 count in the first month of use. However, her stated discontinuation reason was heavy bleeding, with removal within one month of insertion. Another woman also noted weight loss after C-IUD insertion but did not relate it to HIV disease. Similarly, one woman noticed increased respiratory symptoms and had tuberculosis diagnosed following IUC insertion but named bleeding as the main discontinuation reason. Two women requested removal due to concern that IUC use might exacerbate other health conditions, attributed to guidance from health providers.

*‘My reason* (to remove the IUC) *is because I have lot of things like diabetes, high blood* (pressure) *and they complained that my lungs are failing…When they checked my urine, it looks like I’m on my period but, because I'm not menstruating, they said maybe it* (IUC) *does have problems like that. So, I removed it because I wanted to be sure if it's the loop or not'. (8037, LNG-IUS user)*

### Social network perceptions

No participants reported partner or peer inputs as their primary discontinuation reason, but exploring the context around decisions to discontinue the IUC elicited negative perceptions toward the IUC reportedly held by male partners, health care providers, and other influencers within a participant's social network. The earlier description by the participant whose health care provider indicated IUC use could be worsening her chronic health conditions illustrates this nuance to decision-making. Similarly, some of the women requesting IUC removal due to desired fertility mentioned their partners wanting a pregnancy while the women themselves expressed ambivalence. Despite relatively positive personal views, some participants shared less favorable IUC perceptions heard from others. Misperceptions included concerns about cancer or infertility, that the “*womb will come out”* during IUC removal, or a “*baby will come out holding a loop”* if pregnancy occurs while using IUC.

When asked about changes in relationship status relative to IUC use, nine women raised the topic of disclosing IUC use to their partners. While some women said their partners favored IUC use, others mentioned partners' reservations about how the IUC worked, perhaps confusing IUCs with sterilization or hysterectomy.

*‘I told him that I was going to have a loop and he did not have a problem with it. He only asked if it would be possible to have another baby when we want to […] I think he thought it meant removing my womb’. (7054, LNG-IUS user)*

Additional partner reservations included beliefs that IUCs cause “*complications*”, “*tightens*” the womb, or could be felt during sex. A few women alluded to men's concerns about contraception generally, and whether contraceptive use should be disclosed to a partner. One woman's brother equated undisclosed IUC use with witchcraft:

‘*When you are having sex with a man and you have not let them know that you have a loop, they will think that you have something unholy in you*’. (7069, C-IUD user)

Although one C-IUD user suggested that male partners would strongly pressure women to remove the IUC, most said their partners were supportive of IUC use, while two women stated their partner was unaware of their IUC.

### Overall IUC advantages and disadvantages

Women generally appreciated the IUC's contraceptive protection and described IUCs as “safe”, for a “longer duration”, and easy to remove and use correctly. Seven women expressed desire to continue IUC use if not for side effects.

*“If the LOOP did not cause one to menstruate a lot, I would say it is the best method. You don’t have to do anything with it because you know you have it in you.” (7024, C-IUD user)*

Generally, participants liked the multi-year duration and ease of IUC use. However, most (*n* = 12) said they had already resumed, or planned to “*go back*” to injectable use, because they couldn't tolerate excessive bleeding or pain from the IUC. Four women, including three discontinuing the IUC to get pregnant and one who believed pain resulted from incorrect IUC placement, planned to use the IUC postpartum (or in the next month in the last case). Finally, one woman decided sterilization was the best method for her because “*It doesn’t affect the period and I can’t get pregnant”*.

## Discussion

The major themes arising from IUC experiences and discontinuation focused on abdominal or pelvic pain and heavy menstrual bleeding among WLHIV electively discontinuing IUC use while participating in a South African trial. HIV status and provider or partner views had comparatively less influence on discontinuation; this study is among the first qualitative explorations of whether living with HIV contributes to IUC discontinuation decisions. It is notable that the sample sizes for the two methods were different and that few women requested LNG-IUS removal, with the four requesting removal during the 24 month cohort period all interviewed within this sub-study. Given these sample sizes, the ability to make comparisons is limited, though the consensus around bleeding and pain as being both more common experiences and reasons for discontinuation with the C-IUD contextualize the study's higher elective C-IUD removal rates due to pain and heavy bleeding (14), which are similar to that detected in other cohorts (4, 6, 16). Abdominal pain, described as interfering with daily activities, was the highest-ranked discontinuation reason. Regarding heavy bleeding, ranked second highest, women were concerned by blood loss volume and perceived associated health risks, like weight loss.

HIV status had little directly stated impact on perceived IUC safety or discontinuation, though symptoms associated with HIV progression (e.g., tuberculosis diagnosis) following insertion were questioned as attributable to IUC use. Women framed weight loss as a health concern that influenced their decision to discontinue and perceived that weight loss as possibly related to excessive bleeding. However, weight loss may be indirectly related to HIV status by prompting suspicion of HIV infection and attendant stigma within a woman's community though this was not mentioned by the participants and was not specifically probed (17, 18). In light of this finding, standard counselling approaches focused on side-effect expectations and management should be used. Providers and peers similarly had little impact on discontinuation, but misperceptions, like lack of provider knowledge regarding diabetes and IUC use, and negative IUC perceptions might influence continuation indirectly and, importantly, reduce uptake.

Three of four women who discontinued the LNG-IUS experienced minimal or no bleeding and did not describe bleeding changes as problematic. While amenorrhea was not specifically mentioned as a positive feature in these interviews, women in other African settings have perceived amenorrhea to be advantageous (19, 20). A male partners negative perceptions of IUC use did not substantively influence a women's decision to request IUC removal unless pregnancy was desired. Most women had discussed IUC use with their partners, but some mentioned non-disclosure due to reportedly negative views of IUC or contraception overall by male partners, as in other contexts (21, 22). Finally, medical conditions prompted discontinuation in two cases, but the provider's role in recommending or counselling against IUC removal was not probed.

At enrollment, women largely reported injectable contraceptives and/or condoms as their current method (14). While appreciating IUC's longer contraceptive duration, women discontinuing for pain or heavy bleeding were more likely to return to these familiar methods after IUC discontinuation. However, when asked about ideal contraceptive features, many women agreed the IUC was the best method if not for the side effects. Research suggests that women's attitudes towards contraceptive-induced menstrual bleeding changes are highly variable and shaped by individual experiences and social contexts (23). While many women may experience menstrual-related side effects perceived as negative, only a portion discontinue IUCs for this reason (6). Nevertheless, counselling about anticipated side effects including menstrual changes associated with the use of contraceptives has been shown to reduce discontinuation of hormonal methods (24, 25).

The strengths of this study include use of qualitative interviews to probe and prioritize various aspects of discontinuation decision-making, and conducting the interviews among WLHIV to understand the potential impact of HIV on IUC discontinuation in a context of universal ART use. Limitations to this analysis include that only half of women requesting IUC removal agreed to the SSI, possibly resulting in selection bias. Qualitative studies can generally uncover most major themes within a specific demographic with a sample size of 12 interviews (26); there were few LNG-IUS discontinuations in the trial overall so, while all women electively discontinuing the LNG-IUS participated in this sub-study, some themes unique to this group may have been undetected. Because this sub-study did not include women retaining the IUC, whether or how side-effects or motivations for use differed by continuation status cannot be determined. There was limited probing of whether HIV-related factors influenced contraceptive choice and continuation, particularly for LARC methods. Women were invited to make follow-up visits after discontinuation if they experienced persistent problems but were not systematically followed to determine whether side effects attributed to IUC use had resolved. Last, this study is limited to a single setting and sub-group of women who may not be representative of other WLHIV.

In conclusion, this sub-study of WLHIV electively discontinuing IUC use confirmed that pain and/or increased bleeding were the most common reasons for discontinuing the IUC. Misperceptions about IUC at individual, provider, and peer levels may additionally influence IUC discontinuation and should be addressed in provider training and demand generation accompanying expansion efforts.

## Data Availability

The raw data supporting the conclusions of this article will be made available by the authors, without undue reservation.
